# 2-(4-Methoxyphenoxy)-6-methyl-3-oxo-3,6-dihydro-2*H*-pyran-4-yl benzoate

**DOI:** 10.1107/S1600536809004231

**Published:** 2009-02-25

**Authors:** Shi-qiang Yan, Xiao-mei Liang, Jian-jun Zhang, Dao-quan Wang

**Affiliations:** a Department of Applied Chemistry, China Agricultural University, Beijing 100193, People’s Republic of China

## Abstract

The title compound, C_20_H_18_O_6_, has been synthesized from 4-methoxy­phenyl 3-*O*-benzo­yloxy-α-l-rhamnopyran­oside by oxidation on treatment with pyridinium dichromate in the presence of acetic anhydride. In the mol­ecule, the pyran ring adopts an envelope conformation with the O atom at the flap position. Weak inter­molecular C—H⋯O hydrogen bonding is present in the crystal structure.

## Related literature

For general background to enolone derivatives, see: Schmidt *et al.* (1954 ▶); Hodges *et al.* (1963 ▶); Bevan *et al.* (1963 ▶); Ripperger & Seifert (1975 ▶); Yan *et al.* (2008 ▶).
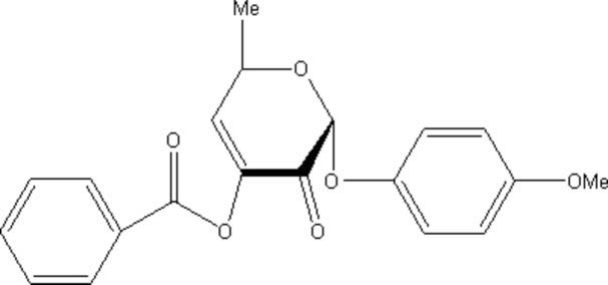


## Experimental

### 

#### Crystal data

C_20_H_18_O_6_
*M* *_r_* = 354.34
Orthorhombic, 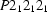

*a* = 8.5906 (17) Å
*b* = 11.594 (2) Å
*c* = 17.404 (4) Å
*V* = 1733.4 (6) Å^3^
*Z* = 4
Mo *K*α radiation
μ = 0.10 mm^−1^
*T* = 173 (2) K
0.80 × 0.72 × 0.40 mm

#### Data collection

Rigaku R-Axis Rapid IP are-detector diffractometer
Absorption correction: multi-scan (*ABSCOR*; Higashi, 1995 ▶) *T*_min_ = 0.924, *T*_max_ = 0.961
3953 measured reflections
2262 independent reflections
1752 reflections with *I* > 2σ(*I*)
*R*_int_ = 0.015

#### Refinement

*R*[*F* ^2^ > 2σ(*F* ^2^)] = 0.034
*wR*(*F* ^2^) = 0.055
*S* = 0.87
2262 reflections
236 parameters
H-atom parameters constrained
Δρ_max_ = 0.17 e Å^−3^
Δρ_min_ = −0.24 e Å^−3^

### 

Data collection: *RAPID-AUTO* (Rigaku, 2001 ▶); cell refinement: *RAPID-AUTO*; data reduction: *RAPID-AUTO*; program(s) used to solve structure: *SHELXTL* (Sheldrick, 2008 ▶); program(s) used to refine structure: *SHELXTL*; molecular graphics: *SHELXTL*; software used to prepare material for publication: *SHELXTL*.

## Supplementary Material

Crystal structure: contains datablocks I, global. DOI: 10.1107/S1600536809004231/xu2467sup1.cif

Structure factors: contains datablocks I. DOI: 10.1107/S1600536809004231/xu2467Isup2.hkl

Additional supplementary materials:  crystallographic information; 3D view; checkCIF report

## Figures and Tables

**Table 1:** Hydrogen-bond geometry (Å, °)

*D*—H⋯*A*	*D*—H	H⋯*A*	*D*⋯*A*	*D*—H⋯*A*
C15—H15*A*⋯O6^i^	0.95	2.42	3.343 (2)	163

Symmetry code: (i) 
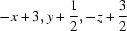
.

**Fig. 1.**
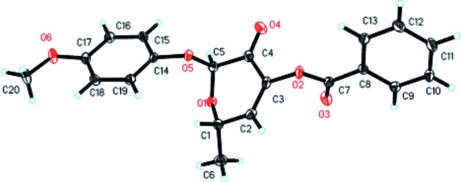
The molecular structure of the title compound, showing the atomic labelling and displacement ellipsoids drawn at the 50% probability level.

## supplementary crystallographic information

### Comment

The enolone structural unit is often present in nature products, such as brevifolic acid, a constituent of the ellagitannins (Schmidt *et al.*, 1954), meliacinslike cedrelone and anthothecol (Hodges *et al.*, 1963; Bevan *et al.*, 1963) or triterpenoids of the elaterin type, which are widely distributed in cucurbitaceous and cruciferous plants (Ripperger & Seifert, 1975). In a continuation of our search for alcohol oxidation (Yan *et al.*, 2008), herein we present the crystal structure of the title compound, which was produced by oxidation with PDC and acetic anhydride.

In the molecule of the title compound (Fig. 1), the pyran ring conformation can be described as an envelope, with C1/C2/C3/C4/C5 lying almost on the same plane and O1 deviating from this mean plane. The terminal benzene rings of the molecule are nearly perpendicular to each other with a dihedral angle of 83.6 (1)°. The weak intermolecular C—H···O hydrogen bonding presents in the crystal structure (Table 1).

### Experimental

A mixture of 4-methoxyphenyl 3-*O*-benzoyloxy-a-*L*-rhamnopyranoside (3.74 g, 10 mmol), pyridinium dichromate (4.60 g, 12 mmol), and acetic anhydride (5.68 ml, 60 mmol) in CH_2_Cl_2_ (40 ml) was stirred at reflux for 8 h, at the end of which time TLC (4:1 petroleumether–EtOAc) indicated that the reaction was complete. After direct concentration of the reaction mixture, the dark brown residue was diluted with EtOAc (60 ml) and the solution was passed through a short (5–10 cm) silica-gel column. The column was eluted with EtOAc and the eluents were concentrated and coevaporated with toluene. The residue was subjected to silica-gel column chromatography again (4:1 petroleum ether–EtOAc) to give the title compound (2.48 g, 70%). Single crystals suitable for X-ray measurements were obtained by recrystallization from 8:1 petroleumether–EtOAc at room temperature.

### Refinement

H atoms were positioned geometrically, with C—H = 0.95 Å, 0.98 Å and 1.00 Å for aromatic, methyl and methine H, respectively, and constrained to ride on their parent atoms, with *U*_iso_(H) = *xU*_eq_(C), where *x*= 1.5 for methyl H and *x* = 1.2 for other H. The absolute structure was not determined for this structure, Friedel pairs were merged.

### Crystal data

**Table:**

C_20_H_18_O_6_	*F*(000) = 744
*M**_r_* = 354.34	*D*_x_ = 1.358 Mg m^−^^3^
Orthorhombic, *P*2_1_2_1_2_1_	Mo *K*α radiation, λ = 0.71073 Å
Hall symbol: P 2ac 2ab	Cell parameters from 789 reflections
*a* = 8.5906 (17) Å	θ = 2.2–27.5°
*b* = 11.594 (2) Å	µ = 0.10 mm^−^^1^
*c* = 17.404 (4) Å	*T* = 173 K
*V* = 1733.4 (6) Å^3^	Block, colorless
*Z* = 4	0.80 × 0.72 × 0.40 mm

### Data collection

**Table:**

Rigaku R-Axis Rapid IP are-detector diffractometer	2262 independent reflections
Radiation source: rotating anode	1752 reflections with *I* > 2σ(*I*)
graphite	*R*_int_ = 0.015
ω scan	θ_max_ = 27.4°, θ_min_ = 2.1°
Absorption correction: multi-scan (*ABSCOR*; Higashi, 1995)	*h* = −11→11
*T*_min_ = 0.924, *T*_max_ = 0.961	*k* = −14→15
3953 measured reflections	*l* = −22→22

### Refinement

**Table:**

Refinement on *F*^2^	Secondary atom site location: difference Fourier map
Least-squares matrix: full	Hydrogen site location: inferred from neighbouring sites
*R*[*F*^2^ > 2σ(*F*^2^)] = 0.034	H-atom parameters constrained
*wR*(*F*^2^) = 0.055	*w* = 1/[σ^2^(*F*_o_^2^)]
*S* = 0.87	(Δ/σ)_max_ = 0.001
2262 reflections	Δρ_max_ = 0.17 e Å^−^^3^
236 parameters	Δρ_min_ = −0.24 e Å^−^^3^
0 restraints	Extinction correction: *SHELXL*, Fc^*^=kFc[1+0.001xFc^2^λ^3^/sin(2θ)]^-1/4^
Primary atom site location: structure-invariant direct methods	Extinction coefficient: 0.0215 (13)

### Special details

**Table:**

Geometry. All e.s.d.'s (except the e.s.d. in the dihedral angle between two l.s. planes) are estimated using the full covariance matrix. The cell e.s.d.'s are taken into account individually in the estimation of e.s.d.'s in distances, angles and torsion angles; correlations between e.s.d.'s in cell parameters are only used when they are defined by crystal symmetry. An approximate (isotropic) treatment of cell e.s.d.'s is used for estimating e.s.d.'s involving l.s. planes.
Refinement. Refinement of *F*^2^ against ALL reflections. The weighted *R*-factor *wR* and goodness of fit *S* are based on *F*^2^, conventional *R*-factors *R* are based on *F*, with *F* set to zero for negative *F*^2^. The threshold expression of *F*^2^ > σ(*F*^2^) is used only for calculating *R*-factors(gt) *etc*. and is not relevant to the choice of reflections for refinement. *R*-factors based on *F*^2^ are statistically about twice as large as those based on *F*, and *R*- factors based on ALL data will be even larger.

### Fractional atomic coordinates and isotropic or equivalent isotropic displacement parameters (Å^2^)

**Table:**

	*x*	*y*	*z*	*U*_iso_*/*U*_eq_	
O1	1.17184 (15)	0.59200 (11)	0.94630 (8)	0.0262 (4)	
O2	0.93224 (17)	0.89681 (11)	0.91264 (8)	0.0298 (4)	
O3	1.04268 (19)	0.94639 (12)	1.02536 (9)	0.0370 (4)	
O4	1.21198 (18)	0.84659 (12)	0.84053 (9)	0.0372 (4)	
O5	1.13746 (15)	0.59345 (11)	0.81269 (7)	0.0230 (3)	
O6	1.36640 (18)	0.16881 (11)	0.72242 (9)	0.0371 (4)	
C1	1.0087 (2)	0.58644 (17)	0.96426 (13)	0.0278 (5)	
H1A	0.9575	0.5302	0.9288	0.033*	
C2	0.9327 (3)	0.70107 (17)	0.95530 (12)	0.0290 (5)	
H2A	0.8318	0.7123	0.9763	0.035*	
C3	1.0009 (3)	0.78794 (17)	0.91900 (12)	0.0257 (5)	
C4	1.1459 (2)	0.77091 (16)	0.87611 (12)	0.0250 (5)	
C5	1.2071 (2)	0.64760 (16)	0.87719 (11)	0.0236 (5)	
H5A	1.3226	0.6488	0.8701	0.028*	
C6	0.9976 (3)	0.5411 (2)	1.04592 (14)	0.0425 (6)	
H6A	1.0486	0.4657	1.0491	0.064*	
H6B	0.8878	0.5333	1.0604	0.064*	
H6C	1.0491	0.5952	1.0810	0.064*	
C7	0.9639 (2)	0.97192 (17)	0.97094 (13)	0.0251 (5)	
C8	0.8888 (2)	1.08488 (16)	0.95760 (12)	0.0230 (5)	
C9	0.8571 (3)	1.15548 (17)	1.02010 (13)	0.0304 (5)	
H9A	0.8852	1.1316	1.0705	0.037*	
C10	0.7844 (3)	1.26088 (18)	1.00880 (15)	0.0379 (6)	
H10A	0.7621	1.3092	1.0515	0.045*	
C11	0.7445 (3)	1.29565 (18)	0.93534 (14)	0.0384 (6)	
H11A	0.6946	1.3679	0.9277	0.046*	
C12	0.7767 (3)	1.22605 (17)	0.87332 (14)	0.0340 (6)	
H12A	0.7498	1.2507	0.8230	0.041*	
C13	0.8478 (2)	1.12062 (16)	0.88406 (12)	0.0278 (5)	
H13A	0.8687	1.0724	0.8412	0.033*	
C14	1.1981 (2)	0.48407 (16)	0.79498 (10)	0.0203 (5)	
C15	1.3486 (2)	0.47295 (16)	0.76804 (11)	0.0242 (5)	
H15A	1.4147	0.5385	0.7652	0.029*	
C16	1.4024 (2)	0.36583 (16)	0.74524 (12)	0.0260 (5)	
H16A	1.5060	0.3573	0.7271	0.031*	
C17	1.3043 (2)	0.27089 (16)	0.74899 (12)	0.0247 (5)	
C18	1.1540 (3)	0.28231 (17)	0.77601 (12)	0.0278 (5)	
H18A	1.0869	0.2173	0.7784	0.033*	
C19	1.1018 (2)	0.39045 (16)	0.79972 (11)	0.0260 (5)	
H19A	0.9992	0.3991	0.8192	0.031*	
C20	1.2665 (3)	0.07108 (16)	0.72123 (15)	0.0446 (7)	
H20A	1.3233	0.0044	0.7010	0.067*	
H20B	1.1765	0.0871	0.6883	0.067*	
H20C	1.2307	0.0544	0.7735	0.067*	

### Atomic displacement parameters (Å^2^)

**Table:**

	*U*^11^	*U*^22^	*U*^33^	*U*^12^	*U*^13^	*U*^23^
O1	0.0262 (8)	0.0268 (8)	0.0256 (8)	0.0052 (7)	−0.0006 (6)	0.0013 (7)
O2	0.0395 (9)	0.0208 (8)	0.0292 (8)	0.0097 (7)	−0.0036 (7)	−0.0066 (7)
O3	0.0455 (10)	0.0285 (9)	0.0370 (10)	0.0032 (7)	−0.0157 (8)	−0.0016 (7)
O4	0.0356 (10)	0.0274 (8)	0.0486 (10)	−0.0030 (8)	0.0019 (8)	0.0054 (8)
O5	0.0236 (8)	0.0212 (7)	0.0244 (7)	0.0041 (7)	−0.0024 (6)	−0.0035 (6)
O6	0.0275 (9)	0.0231 (8)	0.0607 (11)	0.0022 (7)	−0.0003 (9)	−0.0118 (8)
C1	0.0272 (12)	0.0244 (12)	0.0319 (12)	0.0035 (10)	0.0030 (10)	0.0004 (10)
C2	0.0276 (12)	0.0339 (13)	0.0255 (12)	0.0059 (10)	0.0044 (10)	−0.0026 (10)
C3	0.0294 (13)	0.0236 (11)	0.0242 (11)	0.0062 (10)	−0.0048 (10)	−0.0063 (10)
C4	0.0274 (13)	0.0233 (11)	0.0244 (11)	−0.0013 (10)	−0.0071 (10)	−0.0039 (10)
C5	0.0199 (11)	0.0251 (11)	0.0258 (11)	0.0026 (9)	−0.0010 (10)	−0.0013 (10)
C6	0.0481 (15)	0.0426 (14)	0.0367 (14)	0.0099 (13)	0.0097 (12)	0.0110 (12)
C7	0.0249 (12)	0.0227 (11)	0.0278 (12)	−0.0028 (10)	0.0029 (10)	−0.0022 (10)
C8	0.0196 (11)	0.0199 (11)	0.0295 (12)	−0.0031 (9)	0.0026 (9)	−0.0009 (9)
C9	0.0358 (14)	0.0268 (12)	0.0287 (12)	−0.0022 (11)	0.0055 (11)	−0.0010 (10)
C10	0.0484 (16)	0.0218 (11)	0.0434 (15)	0.0026 (12)	0.0142 (13)	−0.0060 (11)
C11	0.0384 (15)	0.0229 (12)	0.0540 (17)	0.0059 (11)	0.0116 (13)	0.0059 (11)
C12	0.0355 (14)	0.0283 (13)	0.0382 (14)	0.0017 (11)	0.0016 (12)	0.0096 (11)
C13	0.0284 (12)	0.0262 (12)	0.0288 (12)	−0.0008 (10)	0.0030 (10)	−0.0023 (10)
C14	0.0229 (12)	0.0194 (10)	0.0185 (10)	0.0038 (9)	−0.0009 (9)	−0.0022 (8)
C15	0.0235 (12)	0.0207 (10)	0.0284 (12)	−0.0047 (10)	0.0006 (10)	0.0005 (9)
C16	0.0170 (11)	0.0284 (12)	0.0326 (12)	0.0022 (9)	0.0041 (10)	−0.0016 (10)
C17	0.0243 (13)	0.0214 (11)	0.0285 (12)	0.0056 (9)	−0.0048 (10)	−0.0028 (9)
C18	0.0236 (12)	0.0232 (11)	0.0367 (13)	−0.0053 (10)	0.0011 (11)	−0.0004 (10)
C19	0.0212 (12)	0.0280 (12)	0.0287 (12)	0.0001 (10)	0.0040 (9)	−0.0019 (10)
C20	0.0363 (15)	0.0194 (11)	0.0782 (19)	0.0025 (11)	−0.0133 (14)	−0.0114 (12)

### Geometric parameters (Å, °)

**Table:**

O1—C5	1.398 (2)	C8—C13	1.391 (3)
O1—C1	1.437 (2)	C9—C10	1.386 (3)
O2—C7	1.365 (2)	C9—H9A	0.9500
O2—C3	1.398 (2)	C10—C11	1.384 (3)
O3—C7	1.201 (2)	C10—H10A	0.9500
O4—C4	1.215 (2)	C11—C12	1.376 (3)
O5—C14	1.405 (2)	C11—H11A	0.9500
O5—C5	1.418 (2)	C12—C13	1.379 (3)
O6—C17	1.378 (2)	C12—H12A	0.9500
O6—C20	1.422 (2)	C13—H13A	0.9500
C1—C2	1.489 (3)	C14—C19	1.367 (3)
C1—C6	1.518 (3)	C14—C15	1.381 (3)
C1—H1A	1.0000	C15—C16	1.383 (3)
C2—C3	1.326 (3)	C15—H15A	0.9500
C2—H2A	0.9500	C16—C17	1.387 (3)
C3—C4	1.465 (3)	C16—H16A	0.9500
C4—C5	1.523 (3)	C17—C18	1.381 (3)
C5—H5A	1.0000	C18—C19	1.394 (3)
C6—H6A	0.9800	C18—H18A	0.9500
C6—H6B	0.9800	C19—H19A	0.9500
C6—H6C	0.9800	C20—H20A	0.9800
C7—C8	1.478 (3)	C20—H20B	0.9800
C8—C9	1.388 (3)	C20—H20C	0.9800
			
C5—O1—C1	114.77 (15)	C10—C9—H9A	120.1
C7—O2—C3	115.67 (16)	C8—C9—H9A	120.1
C14—O5—C5	114.64 (14)	C11—C10—C9	120.0 (2)
C17—O6—C20	117.11 (17)	C11—C10—H10A	120.0
O1—C1—C2	111.40 (17)	C9—C10—H10A	120.0
O1—C1—C6	106.29 (18)	C12—C11—C10	120.3 (2)
C2—C1—C6	112.29 (19)	C12—C11—H11A	119.9
O1—C1—H1A	108.9	C10—C11—H11A	119.9
C2—C1—H1A	108.9	C11—C12—C13	120.2 (2)
C6—C1—H1A	108.9	C11—C12—H12A	119.9
C3—C2—C1	122.3 (2)	C13—C12—H12A	119.9
C3—C2—H2A	118.9	C12—C13—C8	120.1 (2)
C1—C2—H2A	118.9	C12—C13—H13A	120.0
C2—C3—O2	122.5 (2)	C8—C13—H13A	120.0
C2—C3—C4	121.08 (19)	C19—C14—C15	120.83 (18)
O2—C3—C4	116.11 (18)	C19—C14—O5	118.64 (18)
O4—C4—C3	124.03 (19)	C15—C14—O5	120.37 (17)
O4—C4—C5	121.5 (2)	C14—C15—C16	119.60 (18)
C3—C4—C5	114.42 (18)	C14—C15—H15A	120.2
O1—C5—O5	112.68 (15)	C16—C15—H15A	120.2
O1—C5—C4	111.63 (17)	C15—C16—C17	119.73 (19)
O5—C5—C4	105.08 (15)	C15—C16—H16A	120.1
O1—C5—H5A	109.1	C17—C16—H16A	120.1
O5—C5—H5A	109.1	O6—C17—C18	123.95 (19)
C4—C5—H5A	109.1	O6—C17—C16	115.51 (19)
C1—C6—H6A	109.5	C18—C17—C16	120.52 (18)
C1—C6—H6B	109.5	C17—C18—C19	119.18 (19)
H6A—C6—H6B	109.5	C17—C18—H18A	120.4
C1—C6—H6C	109.5	C19—C18—H18A	120.4
H6A—C6—H6C	109.5	C14—C19—C18	120.1 (2)
H6B—C6—H6C	109.5	C14—C19—H19A	119.9
O3—C7—O2	122.79 (19)	C18—C19—H19A	119.9
O3—C7—C8	126.03 (19)	O6—C20—H20A	109.5
O2—C7—C8	111.18 (18)	O6—C20—H20B	109.5
C9—C8—C13	119.72 (19)	H20A—C20—H20B	109.5
C9—C8—C7	119.00 (19)	O6—C20—H20C	109.5
C13—C8—C7	121.27 (18)	H20A—C20—H20C	109.5
C10—C9—C8	119.8 (2)	H20B—C20—H20C	109.5
			
C5—O1—C1—C2	−48.6 (2)	O3—C7—C8—C13	157.1 (2)
C5—O1—C1—C6	−171.17 (16)	O2—C7—C8—C13	−23.2 (3)
O1—C1—C2—C3	14.0 (3)	C13—C8—C9—C10	0.2 (3)
C6—C1—C2—C3	133.1 (2)	C7—C8—C9—C10	−179.0 (2)
C1—C2—C3—O2	−177.84 (19)	C8—C9—C10—C11	−0.3 (3)
C1—C2—C3—C4	8.9 (3)	C9—C10—C11—C12	0.0 (4)
C7—O2—C3—C2	89.0 (2)	C10—C11—C12—C13	0.6 (4)
C7—O2—C3—C4	−97.4 (2)	C11—C12—C13—C8	−0.7 (3)
C2—C3—C4—O4	178.5 (2)	C9—C8—C13—C12	0.3 (3)
O2—C3—C4—O4	4.8 (3)	C7—C8—C13—C12	179.49 (19)
C2—C3—C4—C5	0.3 (3)	C5—O5—C14—C19	116.7 (2)
O2—C3—C4—C5	−173.41 (17)	C5—O5—C14—C15	−67.9 (2)
C1—O1—C5—O5	−59.9 (2)	C19—C14—C15—C16	0.3 (3)
C1—O1—C5—C4	58.1 (2)	O5—C14—C15—C16	−174.99 (17)
C14—O5—C5—O1	−68.5 (2)	C14—C15—C16—C17	0.6 (3)
C14—O5—C5—C4	169.78 (16)	C20—O6—C17—C18	1.8 (3)
O4—C4—C5—O1	149.13 (18)	C20—O6—C17—C16	−176.5 (2)
C3—C4—C5—O1	−32.6 (2)	C15—C16—C17—O6	177.71 (17)
O4—C4—C5—O5	−88.4 (2)	C15—C16—C17—C18	−0.6 (3)
C3—C4—C5—O5	89.8 (2)	O6—C17—C18—C19	−178.37 (19)
C3—O2—C7—O3	−1.6 (3)	C16—C17—C18—C19	−0.2 (3)
C3—O2—C7—C8	178.67 (17)	C15—C14—C19—C18	−1.1 (3)
O3—C7—C8—C9	−23.8 (3)	O5—C14—C19—C18	174.26 (17)
O2—C7—C8—C9	155.93 (18)	C17—C18—C19—C14	1.0 (3)

### Hydrogen-bond geometry (Å, °)

**Table:**

*D*—H···*A*	*D*—H	H···*A*	*D*···*A*	*D*—H···*A*
C15—H15A···O6^i^	0.95	2.42	3.343 (2)	163

Symmetry codes: (i) −*x*+3, *y*+1/2, −*z*+3/2.
